# Sellar repair in endoscopic endonasal transsphenoidal surgery for pituitary adenoma: a report of 240 cases

**Published:** 2012-11

**Authors:** Maryam Jalessi, Guive Sharifi

**Affiliations:** ^*a*^Endoscopic Pituitary and Skull Base Surgery Center, ENT-Head & Neck Research Center and Department, Hazrat Rasoul Akram Hospital, Tehran University of Medical Sciences, Tehran, Iran.; ^*b*^Neurosurgery Department, Loghman Hakim Hospital, Shaheed Beheshti University of Medical Sciences, Tehran, Iran.

## Abstract

**Background:**

Cerebrospinal fluid (CSF) leak is a devastating complication after transsphenoidal surgical approach to pituitary adenoma and sellar repair has been postulated as crucial step in this approach. In this study, we describe our experience regarding sellar repair in pure endoscopic endonasal approach in 240 patients with pituitary adenoma.

**Methods:**

During April 2005 to April 2011, a total of 240 patients with pituitary adenomas underwent endoscopic endonasal approaches for tumor removal. The degree of intra-operative CFS leak was graded as followed: Grade 0: no leakage was observed; grade 1: leakage in form of drops; and grade 3: flow of CSF was observed. Repair was done according to degree of leak e.g. surgicel for grade 0, fat and fascia for grade 1, with adding synthetic sealant and/or lumbar drain for grade 2. The method of repair is discussed in each group and exceptions were also bolded. No sphenoid sinus obliteration was needed.

**Results:**

There were 208 macroadenomas and 32 microadenomas. One hundred and thirteen patients (55.4%) had grade 0 CSF leaks, 78 patients (32.5%) grade 1 CSF leaks, and 29 patients (12%) showed grade 2 leaks. There were 2 documented post operative CSF leak (0.8%) one of them was treated by lumbar drainage and the other with revision endoscopic repair. There was also one case of pneumocephalus (0.4%) with no obvious leak in nasal endoscopic exam which managed medically. There were also 2 cases of post-operation meningitis (0.8%) with no leak that one of them was due to outbreak of acinetobacter in ICU.

**Conclusions:**

Findings of this study showed that intra-operative CSF leak is an important factor determining the need and extend of sellar repair in endoscopic endonasal approach for pituitary adenoma. The low rate of post-operative CSF leak is in favor of the applicability of grading system and method chosen for repair.

**Keywords:**

Sellar repair, Endoscopic endonasal transsphenoidal surgery, Pituitary adenoma,Intra-operative Cerebrospinal fluid leak


					Volume 4, Suppl. 1 Nov 2012
Publisher: Kermanshah University of Medical Sciences
URL: http://www.jivresearch.org
Copyright:
Congress of Iranian Neurosurgeons, 14-16 November 2012, Kermanshah, Iran
Paper No. 68
Sellar repair in endoscopic endonasal transsphenoidal surgery
for pituitary adenoma: a report of 240 cases
Maryam Jalessi a,*
, Guive Sharifi b
a
Endoscopic Pituitary and Skull Base Surgery Center, ENT-Head & Neck Research Center and Department, Hazrat Rasoul
Akram Hospital, Tehran University of Medical Sciences, Tehran, Iran.
b
Neurosurgery Department, Loghman Hakim Hospital, Shaheed Beheshti University of Medical Sciences, Tehran, Iran.
Abstract:
Background: Cerebrospinal fluid (CSF) leak is a devastating complication after transsphenoidal surgical approach to
pituitary adenoma and sellar repair has been postulated as crucial step in this approach. In this study, we describe
our experience regarding sellar repair in pure endoscopic endonasal approach in 240 patients with pituitary
adenoma.
Methods: During April 2005 to April 2011, a total of 240 patients with pituitary adenomas underwent endoscopic
endonasal approaches for tumor removal. The degree of intra-operative CFS leak was graded as followed: Grade
0: no leakage was observed; grade 1: leakage in form of drops; and grade 3: flow of CSF was observed. Repair
was done according to degree of leak e.g. surgicel for grade 0, fat and fascia for grade 1, with adding synthetic
sealant and/or lumbar drain for grade 2. The method of repair is discussed in each group and exceptions were also
bolded. No sphenoid sinus obliteration was needed.
Results: There were 208 macroadenomas and 32 microadenomas. One hundred and thirteen patients (55.4%) had
grade 0 CSF leaks, 78 patients (32.5%) grade 1 CSF leaks, and 29 patients (12%) showed grade 2 leaks. There
were 2 documented post operative CSF leak (0.8%) one of them was treated by lumbar drainage and the other
with revision endoscopic repair. There was also one case of pneumocephalus (0.4%) with no obvious leak in nasal
endoscopic exam which managed medically. There were also 2 cases of post-operation meningitis (0.8%) with no
leak that one of them was due to outbreak of acinetobacter in ICU.
Conclusions: Findings of this study showed that intra-operative CSF leak is an important factor determining the need
and extend of sellar repair in endoscopic endonasal approach for pituitary adenoma. The low rate of post-
operative CSF leak is in favor of the applicability of grading system and method chosen for repair.
Keywords:
Sellar repair, Endoscopic endonasal transsphenoidal surgery, Pituitary adenoma,
Intra-operative Cerebrospinal fluid leak
* Corresponding Author at:
Maryam Jalessi: Endoscopic Pituitary and Skull Base Surgery Center, ENT-Head & Neck Research Center and Department, Hazrat Rasoul Akram
Hospital, Tehran University of Medical Sciences, Tehran, Iran, Tel: 09123000332, Email: mi.shafizad@gmai.com, (Jalessi M.).

				

